# Reporting the epidemiology of aural haematoma in dogs and proposing a novel aetiopathogenetic pathway

**DOI:** 10.1038/s41598-021-00352-0

**Published:** 2021-11-09

**Authors:** Dan G. O’Neill, Yan Hui Lee, Dave C. Brodbelt, David B. Church, Camilla Pegram, Zoë Halfacree

**Affiliations:** 1 Pathobiology and Population Sciences, The Royal Veterinary College, Hawkshead Lane, North Mymms, Hatfield, AL9 7TA Herts UK; 2 Clinical Science and Services, The Royal Veterinary College, Hawkshead Lane, North Mymms, Hatfield, AL9 7TA Herts UK; 3 Davies Veterinary Specialists, Manor Farm Business Park, Higham Gobion, Hitchin, SG5 3HR UK

**Keywords:** Pathogenesis, Epidemiology, Biomechanics

## Abstract

To evaluate the incidence and risk factors for aural haematoma in dogs under primary veterinary care in the UK. A cohort study design. Dogs diagnosed with aural haematoma during 2016 were identified from the VetCompass database. Univariable and multivariable logistic regression modelling were used for risk factor analysis. There were 2,249/905,554 dogs diagnosed with aural haematoma during 2016. The estimated one-year incidence risk for aural haematoma was 0.25% (95% confidence interval 0.24–0.26). After accounting for confounding factors, 14 breeds showed increased odds and 20 breeds showed reduced odds of aural haematoma compared with crossbred dogs. Breeds with the highest odds included Bull Terrier (OR 7.42, 95% confidence interval 4.39–12.54), Saint Bernard (OR 7.28, 95% confidence interval 3.58–14.81) and French Bulldog (OR 6.95, 95% confidence interval 5.55–8.70). Increasing age, increasing bodyweight and breeds with V-shaped drop and semi-erect ear carriage also showed increased odds of aural haematoma. Associations between ear carriage within breeds and the risk of aural haematoma suggest that trauma along the line of cartilage folding within V-shaped and semi-erect ears may trigger aural haematoma. New knowledge of key breed predispositions will contribute to improved breed health control strategies.

## Introduction

Aural haematoma is commonly stated as a frequent diagnosis in veterinary general practice^1–3^ but there is limited population-based information on the precise frequency of aural haematoma in the wider UK canine population. The condition generally presents as a swollen pinna, typically on the inner concave aspect, which is filled with serosanguinous fluid^4,5^. Aural haematoma can cause distress to the dog by pain from the swelling and inflammation of the pinna and also by distress from the added weight of the affected ear^6^.

The pathogenesis of aural haematoma remains unclear, although some studies have documented erosion and fracture of the auricular pinnal cartilage as possible causative factors in dogs with aural haematoma^1,7–9^. A few theories for cartilage damage have been proposed that mainly focus on self-inflicted trauma from head shaking with subsequent blunt force to the pinna^1,8^, or immunological factors^7^. Otitis externa and otitis media have been proposed to lead to head-shaking and therefore to act as predisposing factors for aural haematoma^9–13^. Regardless of aetiology, it is agreed that rupture of the branches of the caudal auricular artery causes blood to pool between the skin on the inside of the pinna and the cartilage^1,5,7,8^. Chronic organisation of the haematoma can lead to fibrosis, contraction and thickening of the pinna, potentially giving a deformed irregular appearance to the affected pinna (‘cauliflower ear’), especially when conservative management is adopted^5,14^.

Few studies have reported on demographic and conformational risk factors for aural haematoma. Some studies have reported an overrepresentation of Labrador Retrievers and Golden Retrievers, and also concurrent otitis externa in dogs diagnosed with aural haematoma^10,15^. A study on 49 affected dogs in Japan reported dogs with advancing age and pendulous ears to be overly represented as aural haematoma cases^15^. However, these studies were limited by relatively small sample sizes and/or did not account for confounding factors by using multivariable analyses. Many of these studies are now quite dated and there have been few recent publications on aural haematoma. The majority of published studies on aural haematoma have focused on descriptive reports regarding the effectiveness of different forms of clinical management^5,11,12,14^. One survey-based study illustrated common treatment methods used by UK primary-care veterinarians^16^. A deeper understanding of the risk factors for aural haematoma could develop new hypotheses on aetiopathogenetic mechanisms for aural haematoma that could be explored further in a priori studies and may also lead to new and more effective therapies or preventive managements.

Using anonymised veterinary clinical data from the VetCompass Programme^17^, this study aimed to report the incidence risk and risk factors for aural haematoma in dogs under primary veterinary care in the UK. The study placed particular focus on associations with breed and conformation. There is currently high interest in understanding associations between breed and conformation with disease in order to identify opportunities to improve the welfare of dogs from individual breeds as well as to support research into the aetiopathogenesis of complex disorders^18^. Based on the previously published results from a small analysis of aural haematoma cases in Japan, the current study hypothesised that breeds with pendulous ears have increased odds of aural haematoma compared to breeds with other types of ear carriage^15^. A secondary aim of the study was to apply the results to generate a more evidence-based theory of the aetiopathogenesis of aural haematoma in dogs. The results of the current study could assist welfare scientists, breeders, veterinary practitioners and owners with an evidence base on the wider general population of dogs to better understand, predict, prevent and manage aural haematoma in dogs. The study did not aim to explore clinical aspects relating to primary veterinary care in any depth.

## Methods

The study population included all available dogs under primary veterinary care at clinics participating in the VetCompass Programme during 2016^17^. Data were extracted on a single year (2016) in order to quasi-standardise the temporal contribution across all the study dogs and therefore to reduce temporal bias^19^. Study designs that include all data on dogs from at any available time tend to bias results towards inclusion of more clinical health data from older dogs that de facto have longer time periods to contribute. Dogs under veterinary care were defined as those with either (a) at least one electronic patient record (EPR), VeNom diagnosis term^20^, free-text clinical note, treatment or bodyweight) recorded during 2016 or (b) at least one EPR recorded during both 2015 and 2017. VetCompass collates de-identified EPR data from primary-care veterinary practices in the UK for epidemiological research^17^. Data fields available to VetCompass researchers include a unique animal identifier along with species, breed, date of birth, sex, neuter status, insurance and bodyweight, as well as clinical information from free-form text clinical notes, summary diagnosis terms and treatment with relevant dates.

A cohort study design was used to estimate the one-year (2016) incidence risk of aural haematoma and to explore associations with demographic and conformational risk factors in this population. Power calculations estimated that a sample of at least 366,454 dogs was needed to estimate incidence risk for a disorder that occurred in 0.25% of dogs with 0.05% acceptable margin of error at a 95% confidence level from a national UK population of 8 million dogs^21,22^. All methods were performed in accordance with the relevant guidelines and regulations. Informed consent for use of the clinical data of the study dogs was obtained from all of the participating clinics and the animal owners. The study is reported in accordance with ARRIVE guidelines^23^.

The case definition for an incident case of aural haematoma required evidence in the clinical records for an aural haematoma event that was first diagnosed during 2016. A new aural haematoma event required at least 4 preceding weeks without evidence of the existence of aural haematoma. It was possible that some of these dogs had been diagnosed with an earlier aural haematoma event greater than 4 weeks previous to the current event. Aural haematoma cases with an aural haematoma episode within the preceding 4 weeks were considering as recurring or ongoing cases and were not included as new events. Diagnostic decision-making was at the discretion of the attending veterinary surgeons. Case-finding involved initial screening of all 905,554 study dogs for candidate aural haematoma cases by searching the clinical free-text field using the search terms aural*, haemat*, hemat* and haematoma ~ 1 with fuzziness to allow 2-letter inversion, insertion or deletion between the date boundaries of July 1st, 2015 and June 30th, 2017^24^. Candidate cases were randomly ordered and the clinical notes of all 4,598 candidate animals were manually reviewed in detail and evaluated for case inclusion. Information was also extracted on concurrent diagnoses of otitis externa and allergic skin disease that were comorbidly reported at the time of first diagnosis of aural haematoma.

Breed descriptive information entered by the participating practices was cleaned and mapped to a VetCompass breed list derived and extended from the VeNom Coding breed list^20^. In the context of this paper, breed was taken to include both recognised purebred breeds and also designer breed terms. A *purebred* variable categorised all dogs of recognisable breeds as ‘purebred’, dogs with contrived names generated from two or more purebred breed terms as designers (e.g. Labradoodle) and dogs recorded as mixes of breeds but without a recorded contrived name as ‘crossbred’^25^. A *breed type* variable included individual breeds (purebreeds or designer crosses) represented by over 3,000 dogs in the overall study population or with ≥ 7 aural haematoma cases, a grouped category of all remaining breed types and a grouping of general crossbred dogs. This approach was taken to facilitate statistical power for the individual breed analyses^26^. A *Kennel Club breed group* variable classified breeds recognised by the UK Kennel Club into their relevant breed groups (Gundog, Hound, Pastoral, Terrier, Toy, Utility and Working) and all remaining types were classified as non-Kennel Club recognised^25^. Breeds were characterised by ear carriage based on pinnal phenotypes typically described for each breed^27–29^. The categories of ear carriage included erect (also known as prick or upright e.g. German Shepherd Dog), semi-erect (also known as cocked or semi-pricked e.g. Rough Collie), V-shaped drop (also known as folded e.g. Hungarian Vizsla), pendulous (also known as drop or pendant, e.g. Basset Hound) and unspecified. Breeds were also characterised by skull shape (dolichocephalic, mesocephalic, brachycephalic, unavailable), haircoat (short, medium, long, unavailable), skull shape (dolichocephalic, mesocephalic, brachycephalic, unavailable), chondrodystrophic (chondrodystrophic, non-chondrodystrophic, unavailable) and spaniel (spaniel, non-spaniel, unavailable) status for analysis.

Neuter and insurance status were defined by the final available EPR value. Adult bodyweight was defined as the mean of all bodyweight (kg) values recorded for each dog after reaching 18 months old and was categorised as: < 10.0, 10.0 to < 15.0, 15.0 to < 20.0, 20.0 to < 25.0, 25.0 to < 30.0, 30.0 to < 40.0 and ≥ 40.0. Mean adult bodyweight was generated for all breed-sex combinations with adult bodyweight available for at least 100 dogs in the overall study population and used to create a variable called ‘bodyweight relative to breed-sex mean’ that categorised individual dogs as “at or above the breed-sex mean”, “below the breed-sex mean” and “unavailable”. Adult bodyweight generated inference on associations with absolute bodyweight while bodyweight relative to breed-sex mean generated inference on associations with the relative bodyweight within breeds. Age (years) was defined at the date of diagnosis of the first 2016 aural hematoma event for cases and at December 31, 2016 for non-cases (i.e. the final date in 2016 that these dogs were not an incident 2016 case) and was categorised as: ≤ 1.0, 1.0 to < 2.0, 2.0 to < 4.0, 4.0 to < 6.0, 6.0 to < 8.0, 8.0 to < 10.0, 10.0 to < 12.0 and ≥ 12.0.

Following internal validity checking and data cleaning in Excel (Microsoft Office Excel 2013, Microsoft Corp.), analyses were conducted using Stata Version 16 (Stata Corporation).

One-year incidence risk with 95% confidence intervals (CI) was described in dogs overall. Incidence risk in 2016 with 95% CI was described in common breeds. The CI estimates were derived from standard errors based on approximation to the binomial distribution^30^. Risk factor analysis used binary logistic regression modelling to evaluate univariable associations between risk factors (*purebred, breed type, Kennel Club recognised breed, Kennel Club breed group, ear carriage, haircoat, chondrodystrophy, skull shape, spaniel, adult bodyweight, bodyweight relative to breed-sex mean, age, sex, neuter* and *insurance*) and aural haematoma during 2016. Because breed was a factor of primary interest for the study, variables that derived from the breed information were excluded from initial breed multivariable modelling because these were highly correlated with breed (*purebred, Kennel Club recognised breed* and *Kennel Club breed group, ear carriage, haircoat, chondrodystrophy, skull shape, spaniel*). Instead, these variables individually replaced the *breed* variable in the main final breed-focused model to evaluate their effects after taking account of the other variables. *Adult bodyweight* (a defining characteristic of individual breeds) replaced *breed* and *bodyweight relative to breed-sex mean* in the final breed-focused model. Risk factors with liberal associations in univariable modelling (*P* < 0.2) were taken forward for multivariable evaluation. Model development used manual backwards stepwise elimination. Clinic attended was evaluated as a random effect and pair-wise interaction effects were evaluated for the final model variables^31^. The area under the ROC curve and the Hosmer–Lemeshow test were used to evaluate the quality of the model fit and discrimination (non-random effect model)^31,32^. Statistical significance was set at *P* < 0.05.

### Ethics approval

Ethics approval was granted by the RVC Ethics and Welfare Committee (reference number reference SR2018-1652).

## Results

### Incidence risk

**Figure 1:**
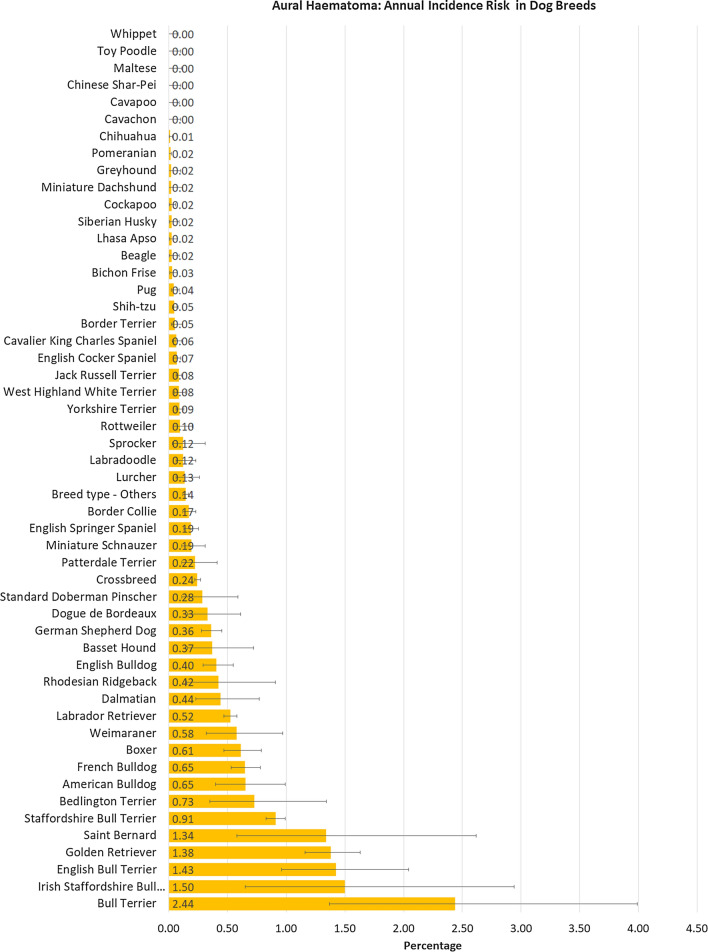
One-year (2016) incidence risk (percentage) for aural haematoma in dog breeds under primary veterinary care in the VetCompass™ Programme in the UK. The horizontal bars represent 95% confidence intervals.

The study included an overall population of 905,554 dogs under veterinary care in 2016 at 887 veterinary clinics. There were 2,249 dogs confirmed with aural haematoma events first diagnosed in 2016 to give an incidence risk during 2016 of 0.25% (95% CI: 0.24–0.26). The breeds with the highest one-year incidence risk for aural haematoma were Bull Terrier (2.44%, 95% CI 1.37–3.99), Irish Staffordshire Bull Terrier (1.50%, 0.65–2.94), English Bull Terrier (1.43%, 0.96–2.04), Golden Retriever (1.38%, 1.16–1.63), Saint Bernard (1.34%, 0.58–2.62) and Staffordshire Bull Terrier (0.91%, 0.83–0.99). Six breeds did not show any cases of aural haematoma: Cavachon, Cavapoo, Chinese Shar-Pei, Maltese, Toy Poodle and Whippet (Fig. 1).

Of the aural haematoma cases with data available for that variable, 1,746 (77.70%) were purebred, 1,118 (49.78%) were female and 1,082 (48.17%) were neutered. Dogs with aural haematoma had a median adult bodyweight of 24.90 kg (IQR: 18.00–32.71, range 1.49–82.00) and median age was 8.17 years (IQR: 5.64–10.47, range 0.16–18.00). The most common breed types among the aural haematoma cases were Staffordshire Bull Terrier (n = 482, 21.43%), crossbred (472, 20.99%), Labrador Retriever (314, 13.96%) and Golden Retriever (135, 6.00%) (Table 1).

**Table 1:** Descriptive and univariable logistic regression results for various dog breeds as risk factors for aural haematoma during 2016 in dogs under primary veterinary care in the VetCompass™ Programme in the UK. Column percentages shown in brackets.

Breed	Case No. (%)	Non-case No. (%)	Odds ratio	95% CI*	Category *P*-value	Variable *P*-value
Crossbreed	472 (0.24)	194,192 (99.76)	1.00	Base		< 0.001
Bull Terrier	15 (2.44)	600 (97.56)	10.29	6.11–17.3	< 0.001	
Irish Staffordshire Bull Terrier	8 (1.50)	525 (98.50)	6.27	3.1–12.68	< 0.001	
English Bull Terrier	29 (1.43)	2005 (98.57)	5.95	4.08–8.68	< 0.001	
Golden Retriever	135 (1.38)	9656 (98.62)	5.75	4.75–6.97	< 0.001	
Saint Bernard	8 (1.34)	589 (98.66)	5.59	2.77–11.29	< 0.001	
Staffordshire Bull Terrier	482 (0.91)	52,567 (99.09)	3.77	3.32–4.28	< 0.001	
Bedlington Terrier	10 (0.73)	1359 (99.27)	3.03	1.61–5.68	0.001	
American Bulldog	21 (0.65)	3202 (99.35)	2.70	1.74–4.18	< 0.001	
French Bulldog	106 (0.65)	16,290 (99.35)	2.68	2.17–3.31	< 0.001	
Boxer	58 (0.61)	9383 (99.39)	2.54	1.93–3.34	< 0.001	
Weimaraner	14 (0.58)	2400 (99.42)	2.40	1.41–4.09	0.001	
Labrador Retriever	314 (0.52)	59,640 (99.48)	2.17	1.88–2.5	< 0.001	
Dalmatian	12 (0.44)	2708 (99.56)	1.82	1.03–3.24	0.040	
Rhodesian Ridgeback	6 (0.42)	1418 (99.58)	1.74	0.78–3.9	0.178	
English Bulldog	38 (0.40)	9361 (99.60)	1.67	1.2–2.33	0.002	
Basset Hound	8 (0.37)	2168 (99.63)	1.52	0.75–3.06	0.242	
German Shepherd Dog	77 (0.36)	21,293 (99.64)	1.49	1.17–1.89	0.001	
Dogue de Bordeaux	10 (0.33)	3022 (99.67)	1.36	0.73–2.55	0.335	
Standard Doberman Pinscher	7 (0.28)	2454 (99.72)	1.17	0.56–2.48	0.675	
Cavapoo	0 (0.00)	4035 (100.00)	1.00	0–0		
Patterdale Terrier	10 (0.22)	4445 (99.78)	0.93	0.49–1.73	0.809	
Miniature Schnauzer	16 (0.19)	8381 (99.81)	0.79	0.48–1.29	0.343	
English Springer Spaniel	38 (0.19)	20,169 (99.81)	0.78	0.56–1.08	0.131	
Border Collie	41 (0.17)	24,347 (99.83)	0.69	0.5–0.95	0.024	
Breed type–Others	122 (0.14)	85,661 (99.86)	0.59	0.48–0.72	< 0.001	
Lurcher	8 (0.13)	6014 (99.87)	0.55	0.27–1.1	0.091	
Labradoodle	9 (0.12)	7476 (99.88)	0.50	0.26–0.96	0.037	
Sprocker	4 (0.12)	3334 (99.88)	0.49	0.18–1.32	0.160	
Rottweiler	7 (0.10)	7278 (99.90)	0.40	0.19–0.83	0.015	
Yorkshire Terrier	25 (0.09)	28,154 (99.91)	0.37	0.24–0.55	< 0.001	
West Highland White Terrier	16 (0.08)	18,862 (99.92)	0.35	0.21–0.57	< 0.001	
Jack Russell Terrier	41 (0.08)	48,529 (99.92)	0.35	0.25–0.48	< 0.001	
English Cocker Spaniel	22 (0.07)	33,051 (99.93)	0.27	0.18–0.42	< 0.001	
Cavalier King Charles Spaniel	11 (0.06)	17,247 (99.94)	0.26	0.14–0.48	< 0.001	
Border Terrier	5 (0.05)	9646 (99.95)	0.21	0.09–0.51	0.001	
Shih-tzu	15 (0.05)	32,895 (99.95)	0.19	0.11–0.31	< 0.001	
Pug	7 (0.04)	16,207 (99.96)	0.18	0.08–0.37	< 0.001	
Bichon Frise	4 (0.03)	13,265 (99.97)	0.12	0.05–0.33	< 0.001	
Beagle	2 (0.02)	8068 (99.98)	0.10	0.03–0.41	0.001	
Lhasa Apso	3 (0.02)	12,546 (99.98)	0.10	0.03–0.31	< 0.001	
Siberian Husky	2 (0.02)	8386 (99.98)	0.10	0.02–0.39	0.001	
Cockapoo	4 (0.02)	18,248 (99.98)	0.09	0.03–0.24	< 0.001	
Miniature Dachshund	1 (0.02)	4827 (99.98)	0.09	0.01–0.61	0.014	
Greyhound	1 (0.02)	5455 (99.98)	0.08	0.01–0.54	0.010	
Pomeranian	1 (0.02)	6220 (99.98)	0.07	0.01–0.47	0.007	
Chihuahua	4 (0.01)	36,790 (99.99)	0.04	0.02–0.12	< 0.001	
Cavachon	0 (0.00)	3535 (100.00)	~	~		
Chinese Shar-Pei	0 (0.00)	3649 (100.00)	~	~		
Maltese	0 (0.00)	3248 (100.00)	~	~		
Toy Poodle	0 (0.00)	3773 (100.00)	~	~		
Whippet	0 (0.00)	4686 (100.00)	~	~		

*CI confidence interval.

Of the dogs that were not aural haematoma cases with data available on the variable, 653,126 (72.57%) were purebred, 430,574 (47.89%) were female and 406,860 (45.26%) were neutered. The median adult bodyweight for non-cases was 13.90 kg (IQR: 8.18–25.00, range 0.72–97.20) and the median age was 4.43 years (IQR: 1.87–8.08, range 0.00–20.97). The most common breeds among the non-case dogs were crossbred (n = 194,192, 21.50%), Labrador Retriever (59,640, 6.60%), Staffordshire Bull Terrier (52,567, 5.82%) and Jack Russell Terrier (48,529, 5.37%) (Table 1).

Otitis externa was concurrently reported in 1,227 (54.6%) of incident aural haematoma cases and allergic skin disease was concurrently reported in 254 (11.3%) of incident aural haematoma cases.

### Risk factors

All variables were liberally associated with aural haematoma in univariable logistic regression modelling and were therefore evaluated using multivariable logistic regression modelling (Tables 1, 2 and 3).

**Table 2:** Descriptive and univariable logistic regression results for breed-derived risk factors for aural haematoma during 2016 in dogs under primary veterinary care in the VetCompass™ Programme in the UK. Column percentages shown in brackets.

Variable	Category	Case No. (%)	Non-case No. (%)	Odds ratio	95% CI*	Category *P*-value	Variable *P*-value
Purebred	Crossbred	472 (0.24)	194,192 (99.76)	Base			< 0.001
	Designer	29 (0.06)	52,657 (99.94)	0.23	0.16–0.30	< 0.001	
	Purebred	1,746 (0.27)	653,126 (99.73)	1.10	0.99–1.22	0.067	
Kennel Club Recognised Breed	Not recognised	547 (0.21)	261,802 (99.79)	Base			< 0.001
	Recognised	1,700 (0.27)	638,173 (99.73)	1.27	1.16–1.40	< 0.001	
Kennel Club Breed Group	Not Kennel Club recognised breed	547 (0.21)	261,802 (99.79)	Base			< 0.001
	Terrier	622 (0.43)	145,292 (99.57)	2.05	1.83–2.30	< 0.001	
	Gundog	543 (0.40)	135,115 (99.60)	1.92	1.71–2.17	< 0.001	
	Working	113 (0.29)	39,102 (99.71)	1.38	1.13–1.69	0.002	
	Pastoral	136 (0.26)	52,845 (99.74)	1.23	1.02–1.49	0.030	
	Utility	207 (0.20)	102,455 (99.80)	0.97	0.83–1.13	0.681	
	Hound	20 (0.06)	31,396 (99.94)	0.30	0.20–0.48	< 0.001	
	Toy	59 (0.04)	131,968 (99.96)	0.21	0.16–0.28	< 0.001	
Ear carriage	Erect	306 (0.19)	162,445 (99.81)	Base			< 0.001
	V-shaped drop	538 (0.44)	123,008 (99.56)	2.32	2.02–2.67	< 0.001	
	Semi-erect	688 (0.35)	196,318 (99.65)	1.86	1.63–2.13	< 0.001	
	Unavailable	503 (0.20)	251,064 (99.80)	1.06	0.92–1.23	0.396	
	Pendulous	214 (0.13)	170,424 (99.87)	0.67	0.60–0.79	< 0.001	
Haircoat length	Short	1,332 (0.39)	338,414 (99.61)	Base			< 0.001
	Medium	347 (0.18)	191,589 (99.82)	0.46	0.41–0.52	< 0.001	
	Long	62 (0.07)	91,961 (99.93)	0.17	0.13–0.22	< 0.001	
	Unavailable	508 (0.18)	281,295 (99.82)	0.46	0.41–0.51	< 0.001	
Skull conformation	Mesocephalic	1,285 (0.31)	416,864 (99.69)	Base			< 0.001
	Brachycephalic	289 (0.17)	166,612 (99.83)	0.56	0.50–0.64	< 0.001	
	Dolichocephalic	172 (0.25)	69,650 (99.75)	0.80	0.68–0.94	0.006	
	Unavailable	503 (0.20)	250,133 (99.80)	0.65	0.59–0.72	< 0.001	
Chondrodystrophic	Not chondrodystrophic	1,417 (0.46)	308,073 (99.54)	Base			< 0.001
	Chondrodystrophic	329 (0.10)	345,012 (99.90)	0.21	0.18–0.23	< 0.001	
	Unavailable	503 (0.20)	250,174 (99.80)	0.44	0.39–0.48	< 0.001	
Spaniel	Non spaniel-type	1,667 (0.29)	576,258 (99.71)	Base			< 0.001
	Spaniel-type	79 (0.10)	76,868 (99.90)	0.36	0.28–0.45	< 0.001	
	Unavailable	503 (0.20)	250,133 (99.80)	0.70	0.63–0.77	< 0.001	

*CI confidence interval.

**Table 3:** Descriptive and univariable logistic regression results for non-breed-related demographic risk factors evaluated for aural haematoma during 2016 in dogs under primary veterinary care in the VetCompass™ Programme in the UK. Column percentages shown in brackets.

Variable	Category	Case No. (%)	Non-case No. (%)	Odds ratio	95% CI*	Category *P*-value	Variable *P*-value
Adult (> 18 months) bodyweight (kg)	< 10.0	145 (0.07)	213,204 (99.93)	Base			< 0.001
	10.0– < 15.0	170 (0.17)	98,218 (99.83)	2.54	2.04–3.18	< 0.001	
	15.0– < 20.0	265 (0.38)	69,122 (99.62)	5.64	4.60–6.90	< 0.001	
	20.0– < 25.0	344 (0.54)	63,555 (99.46)	7.96	6.55–9.67	< 0.001	
	25.0– < 30.0	298 (0.55)	53,472 (99.45)	8.19	6.72–10.00	< 0.001	
	30.0– < 40.0	448 (0.64)	69,477 (99.36)	9.48	7.86–11.44	< 0.001	
	≥ 40.0	170 (0.65)	26,081 (99.35)	9.58	7.68–11.97	< 0.001	
	Unavailable	409 (0.13)	310,130 (99.87)	1.94	1.60–2.34	< 0.001	
Bodyweight relative to breed mean	Lower	765 (0.24)	316,573 (99.76)	Base			< 0.001
	Equal/Higher	1,073 (0.39)	274,419 (99.61)	1.62	1.47–1.78	< 0.001	
	Unavailable	411 (0.13)	312,267 (99.87)	0.55	0.48–0.62	< 0.001	
Age (years)	< 1.0	48 (0.05)	103,820 (99.95)	0.42	0.31–0.58	< 0.001	< 0.001
	1.0– < 2.0	64 (0.05)	130,513 (99.95)	0.45	0.34–0.60	< 0.001	
	2.0– < 4.0	194 (0.11)	178,055 (99.89)	Base			
	4.0– < 6.0	300 (0.21)	139,711 (99.79)	1.97	1.64–2.36	< 0.001	
	6.0– < 8.0	429 (0.38)	112,969 (99.62)	3.49	2.94–4.13	< 0.001	
	8.0– < 10.0	512 (0.56)	90,490 (99.44)	5.19	4.40–6.13	< 0.001	
	10.0– < 12.0	414 (0.63)	65,823 (99.37)	5.77	4.87–6.85	< 0.001	
	≥ 12.0	272 (0.39)	69,466 (99.61)	3.59	2.99–4.32	< 0.001	
	Unavailable	16 (0.13)	12,412 (99.87)	1.18	0.71–1.97	0.518	
Sex	Female	1,118 (0.26)	430,574 (99.74)	Base			0.005
	Male	1,128 (0.24)	468,459 (99.76)	0.93	0.85–1.01	0.074	
	Unavailable	3 (0.07)	4,226 (99.93)	0.27	0.09–0.85	0.025	
Neuter	Entire	1,164 (0.24)	492,175 (99.76)	Base			0.001
	Neutered	1,082 (0.27)	406,860 (99.73)	1.12	1.04–1.22	0.006	
	Unavailable	3 (0.07)	4,224 (99.93)	0.30	0.10–0.93	0.038	
Insurance	Non-insured	1,872 (0.24)	786,076 (99.76)	Base			< 0.001
	Insured	377 (0.32)	117,183 (99.68)	1.35	1.21–1.51	< 0.001	

*CI confidence interval.

**Figure 2:**
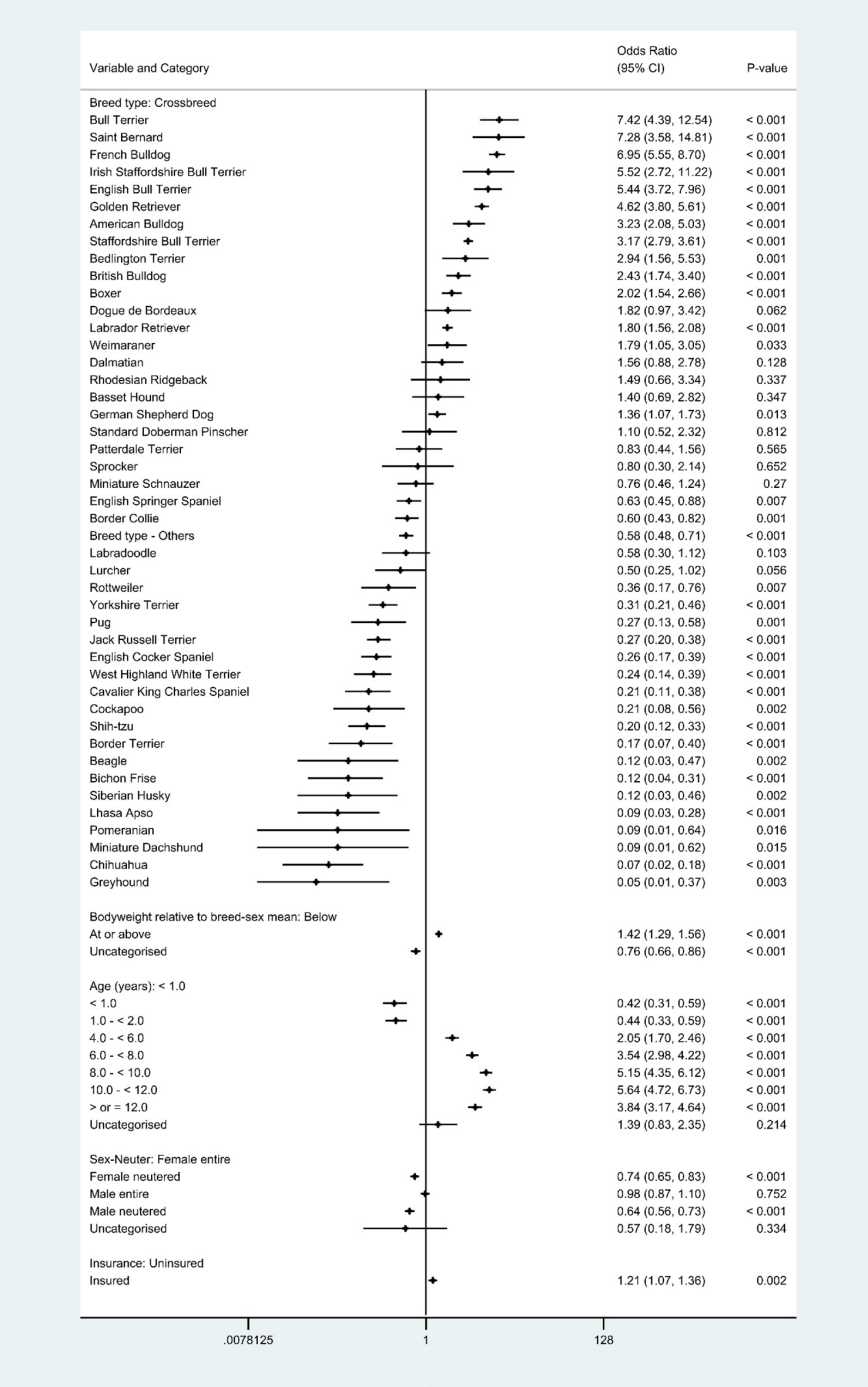
Final breed-focused random effects multivariable logistic regression model for risk factors associated with aural haematoma in dogs under primary veterinary care in the VetCompass™ Programme in the UK. Clinic attended was included as a random effect. *CI confidence interval.

The final main breed-focused multivariable model retained five risk factors: *breed, bodyweight relative to breed-sex mean*, *age, sex-neuter* and *insurance* (Fig. 2). No biologically significant interactions were found in the final models. The final model was improved by inclusion of the clinic attended as a random effect (rho: 0.02 indicating that 2% of the variability was accounted for by the clinic attended, *P* < 0.001). The final model showed acceptable model-fit (Hosmer–Lemeshow test statistic: *P* = 0.232) and good discrimination (area under the ROC curve: 0.828).

After accounting for the effects of the other variables evaluated, 14 breeds showed increased odds of aural haematoma compared with crossbred dogs. Breeds with highest odds included Bull Terrier (odds ratio [OR] 7.42, 95% CI 4.39–12.54, *P* < 0.001), Saint Bernard (OR 7.28, 95% CI 3.58–14.81, *P* < 0.001), French Bulldog (OR 6.95, 95% CI 5.55–8.70, *P* < 0.001), Irish Staffordshire Bull Terrier (OR 5.52, 95% CI 2.72–11.22, *P* < 0.001), English Bull Terrier (OR 5.44, 95% CI 3.72–7.96, *P* < 0.001) and Golden Retriever (OR 4.62, 95% CI 3.80–5.61, *P* < 0.001). Twenty breeds showed reduced odds of aural haematoma compared with crossbreds. Breeds with lowest odds of aural haematoma included Greyhound (OR 0.05, 95% CI 0.01–0.37, *P* = 0.003), Chihuahua (OR 0.07, 95% CI 0.02–0.18, *P* < 0.001), Miniature Dachshund (OR 0.09, 95% CI 0.01–0.62, *P* = 0.015), Pomeranian (OR 0.09, 95% CI 0.01–0.64, *P* = 0.016) and Lhasa Apso (OR 0.09, 95% CI 0.03–0.28, *P* < 0.001). Six breeds had no recorded aural haematoma cases. Dogs with an adult bodyweight that was at or above their breed-sex mean had 1.42 (95% CI 1.29–1.56, *P* < 0.001) times the odds of aural haematoma compared with dogs that weighed below their breed-sex mean. The odds of aural haematoma rose progressively as dogs aged up to 8–12 years and then dropped in dogs above this age. Sex was not associated with the odds of aural haematoma but neutered animals had lower odds than entire animals for both sexes. Insured dogs had 1.21 (95% CI 1.07–1.36, *P* = 0.002) times the odds of aural haematoma compared with uninsured dogs (Fig. 2).

**Figure 3:**
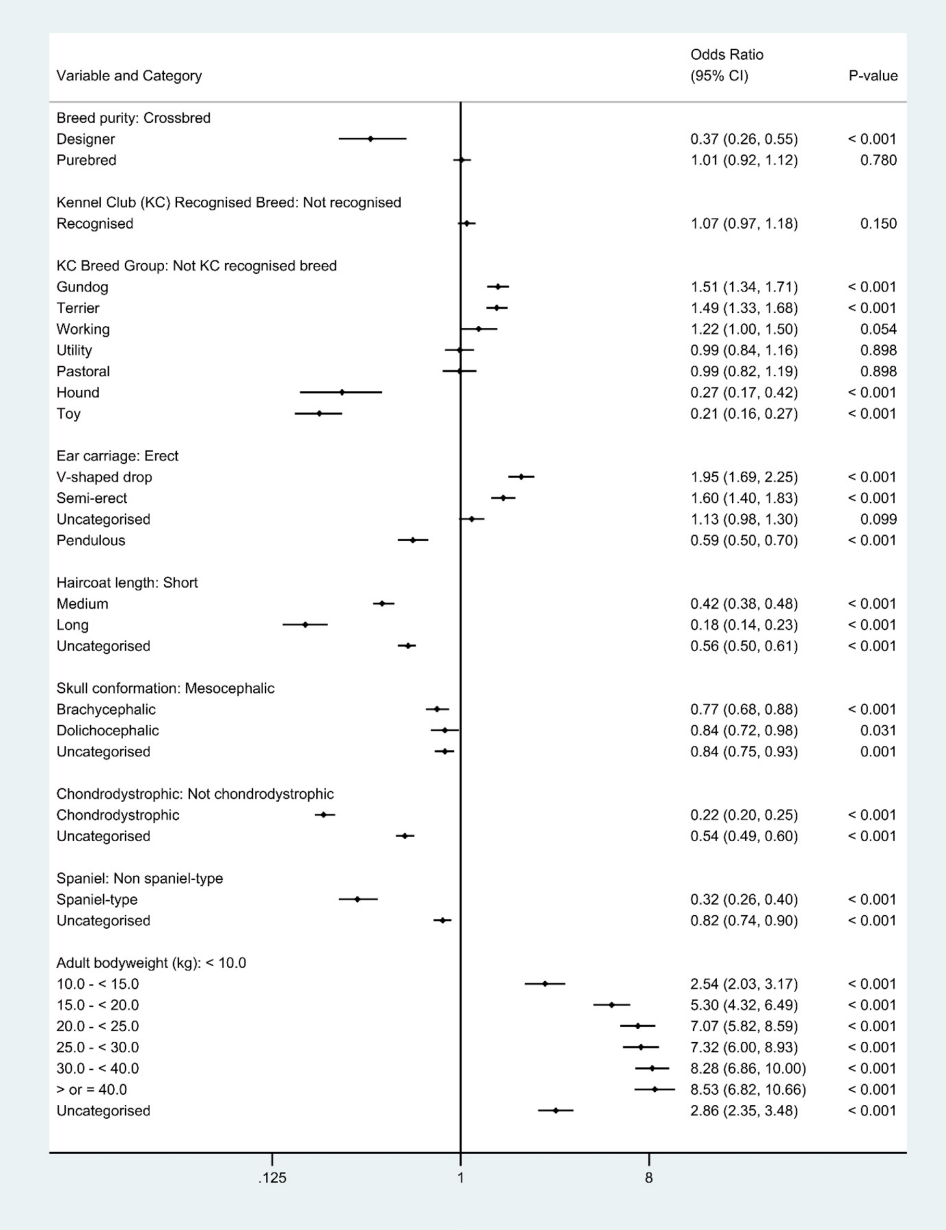
Results for risk factors that directly replaced the breed variable in the final breed-focused mixed effects multivariable logistic regression model (along with age, bodyweight relative to breed-sex mean, sex-neuter and insurance status). These results report associations between these risk factors and aural haematoma in dogs under primary veterinary care in the VetCompass™ Programme in the UK. Clinic attended was included as a random effect. *CI confidence interval.

As described in the methods, breed-derived variables were introduced individually to replace *breed* in the final breed-focused model. Designer breed types had 0.37 times the odds (95% CI 0.26–0.55, *P* < 0.001) of aural haematoma compared with crossbred dogs. Among the Kennel Club breed groups, Gundog and Terrier groups showed higher odds of aural haematoma compared with breeds that are not recognized by the Kennel Club, while Hound and Toy showed lower odds. Breeds with V-shaped drop (OR 1.95, 95% CI 1.69–2.25, *P* < 0.001) and semi-erect ear carriage (OR 1.60, 95% CI 1.40–1.83, *P* < 0.001) had higher odds of aural haematoma compared with breeds with erect ear carriage, while breeds with pendulous ear carriage (OR 0.59, 95% CI 0.50–0.70, *P* < 0.001) had reduced odds. Compared with breeds with short coats, breeds with medium length (OR 0.42, 95% CI 0.38–0.48, *P* < 0.001) and long coats (OR 0.18, 95% CI 0.14–0.23, *P* < 0.001) showed reduced odds of aural haematoma. Compared with breeds with mesocephalic skull conformation, there was reduced odds of aural haematoma in breeds with brachycephalic (OR 0.77, 95% CI 0.68–0.88, *P* < 0.001) and dolichocephalic (OR 0.84, 95% CI 0.72–0.98, *P* = 0.031) skull conformations. Chondrodystrophic breeds (OR 0.22, 95% CI 0.20–0.25, *P* < 0.001) had reduced odds of aural haematoma compared to non-chondrodystophic breeds. Spaniel types had 0.32 times the odds (95% CI 0.26–0.40, *P* < 0.001) of aural haematoma compared with non-spaniel types. The odds of aural haematoma increased as the adult bodyweight of dogs rose (Fig. 3).

## Discussion

Despite being a relatively common diagnosis in general veterinary practice, there is surprisingly little published evidence on the epidemiology of aural haematoma in dogs. This retrospective study characterised the epidemiology of aural haematoma in UK primary-care veterinary practices and the findings are therefore generalisable to the general population of UK dogs. Previous literature had focused predominantly on treatment effectiveness^9,33,34^ while the epidemiology and theories on the aetiopathogenesis of aural haematoma remained unclear^1,7,15,33^. The current study focused on the incidence risk and risk factor associations with aural haematoma in the wider UK dog population using a large data set of dogs under primary veterinary care.

Based on some prior evidence^15^, the current study hypothesised that pendulous-eared breeds had greater odds of aural haematoma compared to breeds with other types of ear carriage. However, the results did not support this hypothesis: breeds with pendulous ears had lower odds than the other types of ear carriage. Conversely, breeds with V-shaped drop ears and semi-erect ears had the highest odds of aural haematoma. This novel finding could be explained by the effects upon the cartilage from the folded structure of the auricular pinnal cartilage: cartilage may be predisposed to fracture at the site of folding. Unlike V-shaped and semi-erect ears that have a degree of rigidity around the ear base and a tendency for the auricular cartilage to fold in the mid-pinnal area, pendulous ears have much greater flexibility and the auricular cartilage within the pinnae does not naturally fold onto itself^27^. Hence, using an inductive approach from the current data, we propose that pendulous ears avoid the trauma from repeated opening and closing of the ear folds that occurs in breeds with V-shaped and semi-erect ears and that this folding may promote fractures of the mid-pinnal cartilage with subsequent predisposition to aural haematoma.

Breed associations with aural haematoma were a primary focus of the current study. There is an important inferential difference for clinicians to note between breeds that contribute a large percentage of aural haematoma cases in veterinary practices (i.e., influenced by proportionately popular breeds and/or frequently affected breeds) and breeds that are intrinsically predisposed to aural haematoma. The relative contribution from differing breeds to the overall caseloads for specific disorders is affected by differing general breed popularity, confounding effects (e.g. age distribution, neuter status differences between these breeds)^35^ and differing innate breed predispositions^36^. This explains why clinicians may perceive that common breeds are more predisposed to disease than rarer breeds and underlines the value of detailed epidemiological analysis based on large populations of representative animals and using multivariable statistical methods for more reliable inference on breed health^36,37^.

The current study provided strong evidence of predisposition to aural haematoma in several breeds. After accounting for other confounding variables, breeds with increased odds of aural haematoma compared with crossbred dogs included the Bull Terrier, Saint Bernard, French Bulldog, Irish Staffordshire Bull Terrier, English Bull Terrier and Golden Retriever. To date, much of the work on dog breed health has focused on identifying predispositions (i.e. increased risk) to diseases^38^. While this information is useful, there is growing interest in identifying which breeds are protected from disease (i.e., reduced risk), because this information on why certain dogs do not get disease may reveal novel insights on aetiopathogenesis and also suggest opportunities for breed reforms to lead to improved health. As a broad concept, widening public awareness of the overall comparative health across dogs breeds could promote a more informed approach to decision-making by owners when selecting a breed to purchase and therefore offers a critical step towards refocusing breed choices based on health rather than aesthetics^39^.

The current study identified that increasing age was associated with rising odds of aural haematoma. Aging is now widely recognised as one of the most important risk factors for many disorders involving degenerative or chronic inflammatory processes^35,40,41^. A study on human auricular cartilage reported increased reorganisation and heterogeneity of elastic fibre diameter with advancing age, suggesting reducing elasticity over time^42^. These findings could mirror changes in dogs, where reduced elasticity may lower the threshold for collagen’s mechanical failure^43^, hence possibly increasing chances of cartilage fracture and aural haematoma. Awareness by owners of age-related increases in risk of aural haematoma may also encourage caution in relation to harsh play activities involving intense head movement in older dogs, e.g., tug-of-war games, to avoid exceeding the threshold for mechanical failure of the auricular collagen. Owners of older dogs could also pay more attention to episodes of intense head shaking or ear scratching and therefore may seek veterinary attention earlier for any subsequent aural haematoma.

Increasing adult bodyweight was associated with rising odds of aural haematoma. Force is defined as mass multiplied by acceleration^44,45^. Based on this law of physics, heavier dogs with their larger head and limb mass are likely to exert much greater centrifugal forces than lighter dogs when they shake their heads either purposefully or during exercise. Thus, heavier dogs are predicted to inflict greater trauma on their pinnae when head shaking, ear scratching or exercising^1,8^. The link between larger physical mass with pinnal centrifugal trauma is supported by the increased risk of aural haematoma within breeds in those subsets of dogs that weigh at or above their breed-and-sex mean bodyweight. Bull breeds such as the English Bulldog and English Bull Terrier breeds were originally selected for larger neck girth and head width to support their roles in bull baiting^25^. Even in the modern examples of these breeds, that are generally household pets rather than working animals, these exaggerated head and neck dimensions may retain increased strength. Predisposition to aural haematoma in these bull breeds lends further support to the theory that either acute or chronic pinnal trauma plays an important role in the aetiopathogenesis of aural haematoma.

Several publications have suggested a causal link between otitis externa and aural haematoma whereby headshaking and ear scratching triggered by otitis externa may lead to pinnal trauma that promotes aural haematoma^9–12,15^. Pendulous ears were previously suggested to predispose dogs to both aural haematoma^46,47^ and otitis externa^48–50^, and this association had been used to explain part of the causal mechanism for otitis externa on aural haematoma. Although there is some evidence that pendulous eared dogs have increased odds of otitis externa^50^, the current study has failed to support the claim that breeds with pendulous ears are predisposed to aural haematoma. An early study on aural haematoma pathogenesis also noted that aural haematoma can occur without detectable inflammatory disease of the ear canal or pinna^1^. Given that 54.82% of aural haematoma cases in the current study were recorded with concurrent otitis externa, it is possible that there are two subsets of dogs with aural haematoma: those that have primary aural haematoma due to chronic pinnal cartilage trauma and those that have secondary aural haematoma following ear scratching and head shaking due to otitis externa. Cohort studies that follow the occurrence of otitis externa in dogs with and without aural haematoma could assist to establish any causal links with aural haematoma. Even if there is an increased incidence of otitis externa in dogs with pendulous ears, these dogs appear protected from developing aural haematoma due to the conformation of the pinnal auricular cartilage.

As with all studies based on the secondary use of veterinary clinical data for research purposes, the current study had some limitations^51^. There was potential misclassification of disorder status due to the secondary use of clinical data. Calculation of age relied on the date of birth information that was recorded in the veterinary clinical records and it is therefore possible that this was an estimated date for many dogs, e.g., dogs that had been rehomed. The noise consequently introduced to the age data may therefore reduce the certainty of the results based on age^52^. However, the level and direction of noise is likely to be similar for both the cases and non-cases so the age-based results are not likely to be substantially biased in one or direction^53^. The current study excluded dogs with aural haematoma that were not documented in EPRs (e.g., an oversight by the attending veterinarian or client did not seek veterinary care for the dog) and hence the true incidence of aural haematoma may be underestimated here. Alternatively, overestimation of aural haematoma was possible because this study did not require aural haematoma aspiration for case confirmation. The analysis based on ear carriage assumed all dogs within each breed had a conformation typical of their assigned ear carriage, but some variation in ear carriage is likely within breeds. Older studies of aural haematoma in the UK may have included dogs with cropped ears. However, although ear cropping of dogs became illegal in England and Wales under Sect. 5 of the Animal Welfare Act 2006 so it is possible the current study still included some imported dogs that may have had cropped ears^54^. The current study focused on epidemiological aspects of aural haematoma and did not aim to identify clinical aspects relating to primary veterinary care beyond comorbidity with otitis externa and allergic skin disease. However, any associations between otitis externa and allergic skin disease with aural haematoma are likely to be highly complex, with differing ages of onset adding a confounding effect to any direct pathophysiological roles that would need to be explored^55^. Future studies could explore differing treatments used for incident and recurrent aural haematoma events, as outlined in a smaller survey-based study^16^.

## Conclusions

This is the largest epidemiological study to date on aural haematoma in the UK dog population and reported an annual incidence risk of 0.25%. Five risk factors were associated with aural haematoma: breed, ear carriage, age, and absolute and relative bodyweight. Bull Terrier, Saint Bernard, French Bulldog, Irish Staffordshire Bull Terrier, English Bull Terrier and Golden Retriever breeds were predisposed to aural haematoma compared with crossbreeds. Dogs with V-shaped drop and semi-erect ear carriage had higher odds of aural haematoma compared with dogs with pendulous ears. A novel theory of trauma along the line of cartilage fold within certain types of ear carriage is proposed as a cause of aural haematoma. These findings of breed and ear carriage associations with aural haematoma will contribute to future understanding of aural haematoma aetiopathogenesis.

## Data Availability

The datasets generated during and/or analysed during the current study are available at the RVC Research Online repository https://doi.org/10.34840/xywc-ew06.

## Abbreviations

CI: Confidence interval
EPR: Electronic patient record
IQR: Interquartile range
IRR: Incidence risk ratio
KC: The Kennel Club
OR: Odds ratio
